# Common Molecular Detection of the Neglected Human Malaria Parasite Among Febrile Patients in Southern Regions in Senegal

**DOI:** 10.3390/pathogens14121201

**Published:** 2025-11-25

**Authors:** Babacar Souleymane Sambe, Serigne Ousmane Mbacké Diaw, Aissatou Diagne, Arona Sabène Diatta, Hélène Ataume Mawounge Diatta, Ibrahima Sarr, Rokhaya Sané, Patindé Yann Bianca Guigma, Bruno Senghor, Babacar Diouf, Papa Mbacke Sembene, Ines Vigan-Womas, Makhtar Niang

**Affiliations:** 1 Pôle Immunophysiopathologie et Maladies Infectieuses, Institut Pasteur de Dakar, Dakar BP 220, Senegal; babacarsouleymane.sambe@pasteur.sn (B.S.S.); ousmanembacke.diaw@pasteur.sn (S.O.M.D.); aissatou.diagne@pasteur.sn (A.D.); arona.diatta@pasteur.sn (A.S.D.); helene.diatta@pasteur.sn (H.A.M.D.); ibrahima.sarr2@pasteur.sn (I.S.); rokhaya.sane@pasteur.sn (R.S.); guigmabianca@yahoo.fr (P.Y.B.G.); bruno.senghor@pasteur.sn (B.S.); babacar.diouf@pasteur.sn (B.D.); ines.vigan-womas@pasteur.sn (I.V.-W.); 2 Département de Biologie Animale, Faculté des Sciences et Techniques, Université Cheikh Anta Diop de Dakar, Fann, Dakar BP 5005, Senegal; mbacke.sembene@ucad.edu.sn

**Keywords:** malaria, *Plasmodium falciparum*, *P. ovale*, *P. vivax*, *P. malariae*, Senegal

## Abstract

Background: In sub-Saharan Africa, *Plasmodium falciparum* is unequivocally responsible for almost all malaria cases and deaths. However, the long-neglected human *P. vivax*, *P. ovale,* and *P. malariae* parasites also emerge as relevant, though their prevalence and contribution to the burden of the disease are very poorly appreciated. This study aimed to bridge this gap and surveyed the circulation of non-falciparum malaria parasites among febrile patients in four regions in south Senegal. Methods: Blood samples were obtained from 1990 febrile patients during the malaria transmission seasons of 2020, 2021, and 2022 in four southern regions in Senegal (Kedougou, Kolda, Tambacounda, and Ziguinchor). Genomic DNA was isolated and tested for *Plasmodium* infections by using a combination of *Plasmodium* genus-specific qPCR and *Plasmodium* species-specific nested PCR. Frequencies and distribution of *Plasmodium* species according to region, period, and patient demographics were analyzed using R. Spatial patterns of infection were further explored and visualized with QGIS software version 3.30.2. Results: The *Plasmodium* positivity rate was 73.43% of which 67.92% were unique *Plasmodium* species infections and 32.08% were co-infections by two or three *Plasmodium* species. The results described the ongoing circulation of all non-falciparum species in three of the four study regions, the non-detection of *P. vivax* and *P*. *malariae* parasites among the samples tested in Ziguinchor, the first evidence of non-falciparum infections in Kolda and Tambacounda, as well as the first report of *P. ovale* in Ziguinchor. Conclusions: Our data call on clinicians to account for these species in clinical prognoses, but also on the National Malaria Control Programme to consider these species in their policy of reducing the incidence of the disease with a view to eliminating malaria in Senegal.

## 1. Introduction

Malaria infection rates vary widely among populations, and children younger than 5 years and primigravidae pregnant women in the WHO African region are the most affected individuals [1]. As the main cause of malaria in Africa, *Plasmodium falciparum* accounts for more than 90% of the world’s malaria mortality, thus justifying that the majority of preventive and control measures against the disease are directed towards this species.

Though there is a renewed interest in human-infecting non-falciparum species in sub-Saharan Africa, the scarcity of research on their prevalence and role in malaria disease have somewhat limited the attention paid to these species. *Plasmodium vivax* is well acknowledged to pose a huge threat to human health [2], but the contribution of other non-falciparum species, notably *P. ovale* and *P. malariae*, to the burden of the disease remains very poorly documented. Despite being incriminated for disease severity and deaths in some studies [3,4,5,6,7], many reasons contribute to explain the poor understanding of the role of non-falciparum species in malaria morbidity and mortality. These include, but are not limited to, the absence of validated sensitive and species-specific point-of-care diagnostics, their co-occurrence with *P. falciparum* at low parasitemia levels, and the lack of experienced microscopists to distinguish between *Plasmodium* species. Moreover, the ability of *P. vivax* and *P. ovale* to constitute dormant hypnozoites forms that can revive infections in the absence of new mosquito bites, along with the inability to maintain both species in long-term in vitro culture because of their specific biological features, constitute additional challenges to the assessment of their contribution to the burden of malaria.

In Senegal, the National Malaria Control Programme (NMCP) has reported, despite a substantial regional variation, a global trend of decreased malaria-related morbidity and mortality in the last two decades. In fact, the NMCP data indicate that in the southern regions, the pattern of malaria transmission varies from high in the southeast (Kedougou, Kolda, and Tambacounda), moderate in the central (Sedhiou), to low in the southwest (Ziguinchor) regions [8]. The NMCP routine surveillance of malaria uses the standard of care malaria Rapid Diagnostic Test (RDT) and microscopy methods for the diagnosis of malaria cases and identifies *P. falciparum* as the major cause. These make *P. falciparum* the primary target for the control and elimination of the disease, though a low-level of non-falciparum species (*P. ovale*, *P. vivax,* and *P. malariae*) infections is recurrently reported [7,9,10,11,12,13,14]. Although effective and inexpensive, RDT and microscopy methods lack sufficient sensitivity to identify non-falciparum species, which further drops with the decrease in parasitemia during the disease elimination phase. Molecular methods are now widely used for the detection and identification of malaria parasites, including in mixed infections and at low parasitemia [15,16].

This study surveyed the circulation of the neglected non-falciparum human malaria parasites among febrile patients living in southern regions of Senegal.

## 2. Materials and Methods

### 2.1. Study Sites

**Figure 1:**
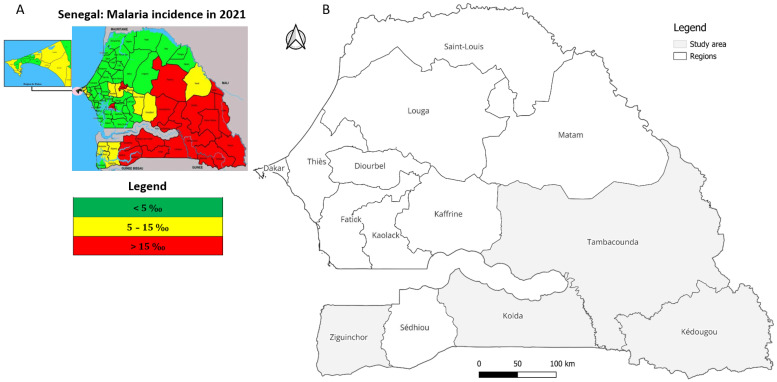
Map of Senegal showing (**A**) the highest malaria incidence regions (red) in the south and southeast in 2021, adapted from an NMCP report [8] and (**B**) a highlight of the four selected study regions.

This study was conducted in four of the fourteen regions of Senegal situated in the south-eastern and south-western parts of the country (Figure 1). These include Kedougou (12°32′58″ N, 12°10′58″ W), Kolda (12°53′04″ N, 14°55′11″ W), Tambacounda (13°45′12″ N, 13°40′18″ W), and Ziguinchor (12°33′29″ N, 16°16′56″ W) [17].

A detailed description of Kedougou region (climate, rainfall, landscape, fauna, population, malaria prevalence) has been provided elsewhere [18,19,20]. Tambacounda region, located in the southeast, is the largest region of Senegal, bordered by The Gambia, Guinea, and Mali. The rainy season runs from June to November and the majority of malaria cases occur between July and October [20]. The region of Kolda is located in the south, bordered by The Gambia in the north, Guinea-Bissau and Guinea in the south, Sédhiou region in the west, and Tambacounda in the east. Ziguinchor region is located in the southwest of the country, bordered by The Gambia in the north and Guinea-Bissau in the south. It is the eighth largest region of Senegal, and is geographically isolated from the north of the country by The Gambia. The region has a tropical savanna climate, with an average annual accumulated rainfall of approximately 1547 mm or 61 inches.

According to recent data from the NMCP [8], Senegal recorded a total of 354,708 malaria cases. Of these, 116,983 cases (32.98%) were reported in Kolda, 101,077 (28.49%) in Tambacounda, 67,941 (19.15%) in Kédougou, and 3757 (1.06%) in Ziguinchor. Together, these regions account for approximately 81.69% of the country’s total malaria burden.

### 2.2. Study Design and Sample Collection

This study focused on febrile patients who consulted at the regional hospitals in Tambacounda, Kolda, and Ziguinchor, while in Kedougou patients were recruited from the primary health center and the health posts of Bandafassi, Bantako, and Ndiormi. Patients were enrolled during the malaria peak transmission seasons (from August to December) in 2020, 2021, and 2022. All patients aged 6 months and older presenting with fever (axillary temperature > 37.5 °C) and at least one symptom suggestive of malaria disease (headache, nausea, dizziness, chills, fatigue, etc.) were included in the study after obtaining their consent or assent.

A total of 1990 patients were included in the study, and venous blood samples were collected on EDTA tubes from each participant along with age and sex demographic data.

### 2.3. Molecular Characterization of Malaria Parasite Species

All blood samples were subjected to *Plasmodium* species characterization following genomic DNA (gDNA) extraction using a QIamp DNA Blood Mini Kit (Qiagen, Hilden, Germany) according to the manufacturer’s instructions. A *Plasmodium* genus-specific qPCR assay targeting the cytochrome b [21] was used for the molecular detection of species-specific *Plasmodium* parasite DNA, followed by a nested PCR on qPCR positive DNA with primers targeting the *Plasmodium* spp. 18S small subunit ribosomal RNA (18S ssrRNA) genes as described previously [15]. Genomic DNA obtained from samples found positive by microscopy for *P. falciparum*, *P. malariae*, *P. vivax,* and *P. ovale*-infected patients were used as positive controls. Nested PCR results were scored as a categorical variable (presence versus absence of amplification).

### 2.4. Data Analysis

The structure of the study population was analyzed according to sex and age of patients, and their distribution according to three defined age groups: <5 years, 5–15 years, and ≥15 years. The frequency of the different *Plasmodium* species was determined by calculating the proportions of each species and each type of infection (single or mixed). The significance of the observed differences was assessed using Fisher’s exact test for categorical variables or the Kruskal–Wallis test for comparison of means. All statistical analyses were performed using R version 4.1 [22] with a significance level of 5% and all maps were realized using QGIS version 3.30.2 [23].

## 3. Results

### 3.1. Demographic Characteristics of the Study Population

A total of 1990 patients were included in the study, and their demographic characteristics are summarized in Table 1. Of these, only 7.9% originated from Ziguinchor while the majority (47.1%) were recruited in Tambacounda, the region with the lowest mean age (17.29 ± 0.56) of participants as well as the youngest population (60.9% were less than 15 years old) (Table 1). Males and females were equally represented in Tambacounda, Kedougou, and Ziguinchor, whereas male participants predominated in Kolda (Table 1). Patients aged +15 years old constitute the majority of participants irrespective of the region (Table 1).

### 3.2. Plasmodium Species Infections Among the Study Patients

*Plasmodium* genus positivity among the enrolled patients was 68.69% (1367/1990) as measured by qPCR, and all the four *Plasmodium* species tested in this study were detected among the patients’ samples with *P. falciparum* being the most dominant parasite (63.82%) (Table 2). Among the neglected non-falciparum parasites, *P. ovale* was detected in 41.61% of patients’ samples (Table 2), while only 2.7% (54/1990) and 2.06% (41/1990) of samples tested positive for *P. vivax* and *P. malariae,* respectively (Table 2).

All *Plasmodium* species were detected in both male and female patients, but a statistically significant difference was only noted for *P. falciparum* (*p*-value = 0.04) (Table 2). With regards to age, besides *P. malariae* (*p* = 0.8), the other three *Plasmodium* species showed significant differences between age groups, with patients aged ≤15 years being more infected than the others (*p* < 0.05) (Table 2).

### 3.3. Single and Mixed Plasmodium Species Infections Among Patients

**Figure 2:**
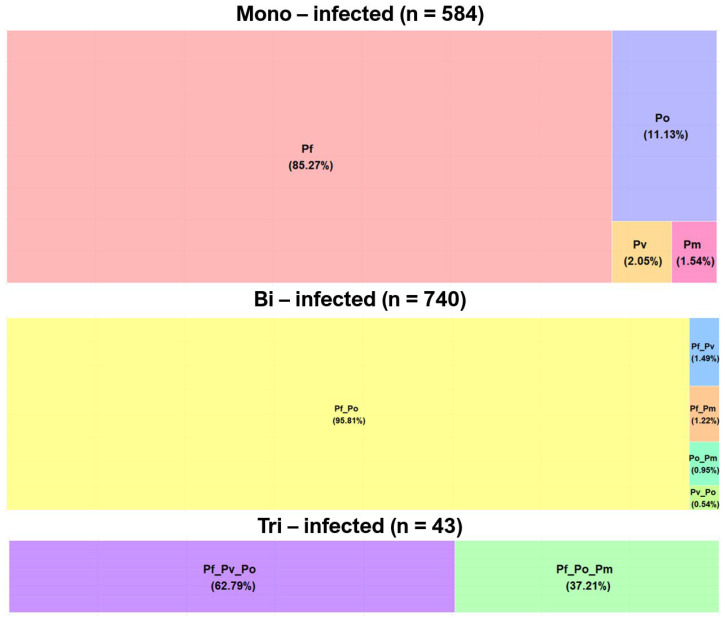
Single and mixed *Plasmodium* species infections among the study samples (Pf: *Plasmodium falciparum*; Pv: *P. vivax*; Po: *P. ovale*; Pm: *P. malariae*).

Patients mono-infected with *Plasmodium* species accounted for 42.7% (584/1367) of all detected *Plasmodium* infections (Figure 2). Among the 584 mono-infected samples, close to 15% were due to non-*falciparum* parasites at the respective proportions of 11.13%, 2.05%, and 1.54% for *P. ovale*, *P. vivax*, and *P. malariae* (Figure 2).

Of the 740 bi-infected patients, 95.8% were co-infected by *P. falciparum*/*P. ovale* followed by *P. falciparum*/*P. vivax* (1.49%), *P. falciparum*/*P. malariae* (1.22%), *P. ovale*/*P. malariae*: 0.95%), and *P. ovale*/*P.vivax* (0.54%). Among the 43 samples concurrently infected by three *Plasmodium* species, only two combinations (*P. falciparum*/*P. vivax*/*P. ovale* and *P. falciparum*/*P. ovale*/*P. malariae*) were detected at 62.79% and 37.21%, respectively (Figure 2).

The specific frequencies of single and mixed non-*Plasmodium* species infections are detailed in Supplemental Material S1 for each region.

### 3.4. Spatio-Temporal Variation of Non-Falciparum Species Infections

**Figure 3:**
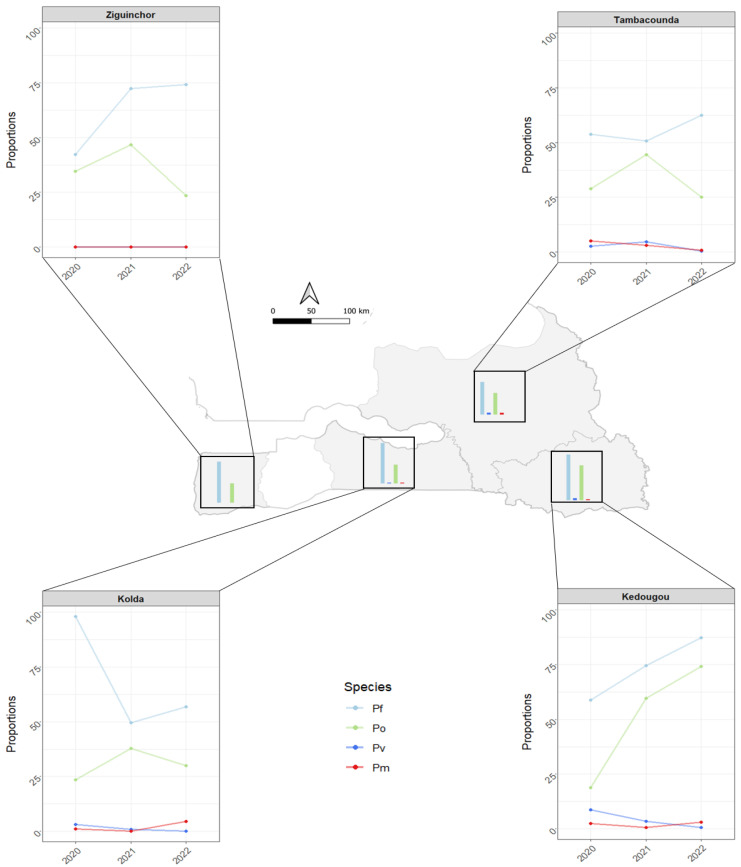
Spatial and temporal distribution of *Plasmodium* species in the four southern regions of Senegal from 2020 to 2022 (Pf: *Plasmodium falciparum*; Pv: *P. vivax*; Po: *P. ovale*; Pm: *P. malariae*).

The proportions of *Plasmodium* species varied between the study regions and sampling periods (2020, 2021, and 2022) and are illustrated in Figure 3. In Ziguinchor, *P. ovale* was the only non-*falciparum* species detected among the tested samples. This contrasts with the detection of the three non-falciparum parasites across the study periods in all the three other study regions (Kedougou, Tambacounda, and Kolda), with *P. ovale* being the most represented while *P. vivax* and *P. malariae* were found at lower and comparable proportions (Figure 3 and Supplemental Material S2).

Between the three study periods, only *P. ovale* has shown substantial variation in frequency in all regions, a finding that contrasts with the low frequencies and limited variation of *P. vivax* and *P. malariae* in Tambacounda, Kedougou, and Kolda (Figure 3). The variation pattern of *P. ovale* was similar in Ziguinchor, Kolda, and Tambacounda, and was characterized by an important frequency increase from 20–30% in 2020 to 37.5–45% in 2021 and a subsequent drop close to 25% in 2022 (Figure 3). In Kedougou, the proportion of *P. ovale* increased from 18.75% in 2020 to 60% in 2021, and 74.21% in 2022 (Figure 3).

## 4. Discussion

In Senegal, the malaria incidence data reported by the NMCP are solely based on *P. falciparum*-based RDT and microscopic detection of *Plasmodium* species, both being indeed of great use in remote areas that lack modern laboratory capabilities. However, both methods have shown several limitations in the detection of non-falciparum species, thus hampering the estimation of the true prevalence and contribution of non-falciparum species to the global malaria burden [24]. To end malaria, the detection and clearance of all malaria parasites that affect humans are critical, and molecular assays are keys to this perspective, not only by confirming the high *P. falciparum* malaria incidences in various settings, but also by revealing instances of non-falciparum infections that have surfaced in many sub-Saharan African countries [16,25,26,27,28,29], including in Senegal [7,11,18,30]. However, it is worth noting that a selection bias of patient recruitment in 2020 in Kolda has resulted in the high frequency of *P. falciparum* infections in that area, but similar trends and infection rates were observed in 2021 and 2022 in all regions.

Besides the absence of *P. vivax* and *P. malariae* parasites in patients’ samples obtained from Ziguinchor, this study has revealed the presence of all non-falciparum parasites in samples from Kedougou, Tambacounda, and Kolda across the three periods. The results confirm early reports on the circulation of non-falciparum species in Kedougou [7,11,18,30], but, to the best of our knowledge, report the first detection of *P. ovale* parasite infections in Ziguinchor.

In this study, non-falciparum species were predominantly found in co-infections with *P. falciparum* at varying proportions across the four regions, an association that further complicates differentiation by microscopy [31,32] in areas where technicians are not sufficiently trained. Living in co-endemic areas with active transmission of all human non-falciparum species was shown to be predictive of exposure to infection by one malaria species with an increased risk of exposure to another malaria species [33]. This finding underscores the need for an improved surveillance system with quality-assured diagnosis by coupling the current standard point-of-care diagnostics with molecular surveillance to sustain *Plasmodium* species detection and speciation. The high proportion of younger patients (60.9% <15 years) from Tambacounda may explain the high *Plasmodium* infection rate among this more vulnerable population, as opposed to Ziguinchor, which has the highest proportion of older patients (81.63% ≥15 years) who are known to be less vulnerable to *Plasmodium* infections and malaria disease.

Though the majority of *P. vivax* and *P. ovale* infections are reported to originate from relapses of dormant hypnozoites parasites from the liver [34], we cannot, at this stage, argue about whether the PCR-detected *P. ovale* and *P. vivax* parasites were new infections from recent mosquito bites or were due to recrudescence from the dormant hypnozoites parasites of old infections. The data indicate that non-falciparum *Plasmodium* species circulate in the study areas, and their low-level detection among patients who consulted at given health facilities suggests the potential existence of an important reservoir in the communities. Similar observations have been previously made, and have raised the question of the existence of potential “hot/cold” spots of transmission that require further investigations if the elimination objective is to be achieved in due time [35]. The exclusion of P. knowlesi, the fifth human-infecting Plasmodium species from our molecular panel is justified by the lack of epidemiological evidence to suspect it’s introduction in Senegal. However, we recognize the importance of proactive surveillance, especially in the context of changing ecological dynamics, increasing human–animal interactions, and mosquito vector adaptation that could facilitate zoonotic transmission.

The control of life-threatening infections due to non-falciparum species (*P. ovale*, *P. vivax,* and *P. malariae*) is a major obstacle to malaria elimination [36], because of several factors: the diagnostic challenge posed by their low-density parasitemia; the ability of *P. vivax* and *P. ovale* to revive infections from dormant hypnozoites forms; and the inability to maintain them in long-term in vitro culture [36,37,38] for advanced experimental studies. Future studies that integrate vector surveillance and environmental factors as well as patients’ clinical data would allow a more detailed analysis and explanation of any potential regional and spatial difference in *Plasmodium* species prevalence and distribution including malaria transmission dynamics and the role of specific Anopheles species in the maintenance of the observed infections. To date, maintaining the limited success gained in controlling *P. falciparum* malaria in the highest malaria transmission regions (Kedougou, Kolda, and Tambacounda) is likely to be challenged by the growing evidence of non-falciparum infections in the areas, thus bringing in a new paradigm for the control program that might require immediate attention. In this context, control and surveillance programs must not only adapt to address the diversity of human *Plasmodium* species but should also, within a proactive surveillance framework, include emerging zoonotic threats such as *P. knowlesi*, which is increasingly recognized for its potential for human transmission.

## 5. Conclusions

This study demonstrates the circulation of the neglected non-*falciparum* malaria parasites among febrile patients in Senegal along with the first evidence of *P. ovale* infections in the low transmission area of Ziguinchor. Though the primary role of *P. falciparum* in malaria incidence is unequivocal, the recurrence of non-falciparum malaria is increasingly becoming a major recognized and neglected public health problem that may threaten the prospect of malaria elimination in Senegal and elsewhere. To further reduce malaria and eliminate the disease, it is urgent to implement malaria molecular surveillance for improved detection of malaria parasites and proper management of malaria patients. The so-called “minor” or “neglected” malaria species deserve special attention as they can cause severe malaria and may lead to fatalities.

## Figures and Tables

**Table 1:** Baseline characteristics of the study population.

	Tambacounda	Kedougou	Kolda	Ziguinchor
Effective	938	594	300	158
Age (years)				
Mean ± SE	17.29 ± 0.56	20.93 ± 0.62	26.72 ± 1.21	34.13 ± 1.67
Median	12	18	22	29
Range	0.5–99	0.5–100	0.5–83	3–98
NA’s number	10	2	8	11
Age groups (%)				
[0–5[ years	28.88	10.64	14.73	2.72
[5–15] years	32.11	30.07	23.97	15.65
15+ years	39.01	59.29	61.3	81.63
NA’s number	10	2	8	11
Sex (%)				
Female	49.09	49.49	43.29	47.47
Male	50.91	50.51	56.71	52.53
Sex ratio (M/F)	1.04	1.02	1.31	1.11
NA’s number	9	0	2	0

SE: Standard error.

**Table 2:** Prevalence of *Plasmodium* species in relation to sex and age.

		Pf	Pv	Po	Pm	Global
Number of infections		1270	54	828	41	1367
Frequency (%)		63.82	2.71	41.61	2.06	68.69
Sex (%)						Effective
	F	61.74	2.41	40.67	1.89	954
	M	66.05	3.02	42.44	2.15	1025
	*p*-value	0.049	0.412	0.438	0.750	-
Age (%)						
	[0–5] years	65.34	3.97	45.5	1.85	378
	[5–15] years	71.00	3.34	45.87	1.76	569
	15+ years	59.49	1.88	37.94	2.27	1012
	*p*-value	<0.001	0.045	0.002	0.810	-

Pf: Plasmodium falciparum; Pv: *P. vivax*; Po: *P. ovale*; Pm: *P. malariae*; SE: Standard error.

## Data Availability

The datasets supporting the conclusions of this article are included within the article. The data supporting the conclusions of this article can be made available by the authors under reasonable request.

## Supplementary Materials

The following supporting information can be downloaded at: https://www.mdpi.com/article/10.3390/pathogens14121201/s1.
