# Supplementary material for: Common Molecular Detection of the Neglected Human Malaria Parasite Among Febrile Patients in Southern Regions in Senegal
Source: Pathogens. 2025 Nov 25;14(12):1201. doi: 10.3390/pathogens14121201 (PMC12735732; doi:10.3390/pathogens14121201)
Supplement: Supplementary file 1 [file pathogens-14-01201-s001.zip › pathogens-3731545_Supplemental Material S2.pdf]

Supplemental Material S2: Prevalence of individual malaria parasite species according to site and year.

	Pf	Pv	Po	Pm
**Kedougou**	**75.93**	**3.37**	**58.80**	**1.52**
2020	58.75	8.75	18.75	2.50
2021	74.65	3.38	59.72	0.56
2022	87.42	0.63	74.21	3.14
**Kolda**	**67.00**	**1.33**	**31.33**	**1.33**
2020	97.96	3.06	23.47	1.02
2021	49.63	0.74	37.78	0.00
2022	56.72	0.00	29.85	4.48
**Tambacounda**	**54.35**	**3.20**	**36.03**	**2.99**
2020	53.85	2.71	28.96	4.98
2021	50.72	4.74	44.54	3.09
2022	62.50	0.43	25.00	0.86
**Ziguinchor**	**68.35**	**0.00**	**32.03**	**0.00**
2020	42.31	0.00	34.62	0.00
2021	72.34	0.00	46.81	0.00
2022	74.12	0.00	23.53	0.00

Pf: *Plasmodium falciparum*; Pv: *P. vivax*; Po: *P. ovale*; Pm: *P. malariae*
