# Supplementary figures and images for: Common Molecular Detection of the Neglected Human Malaria Parasite Among Febrile Patients in Southern Regions in Senegal
Source: Pathogens. 2025 Nov 25;14(12):1201. doi: 10.3390/pathogens14121201 (PMC12735732; doi:10.3390/pathogens14121201)

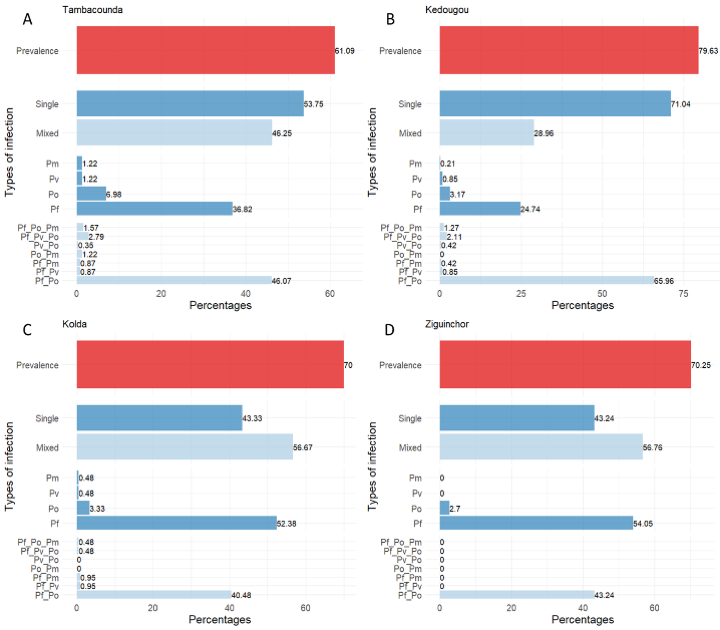

Supplement: Supplementary file 1 [file pathogens-14-01201-s001.zip › pathogens-3731545_Supplemental Material S1.tiff]
